# Pediatric Neurology Briefs: Year in Review

**DOI:** 10.15844/pedneurbriefs-32-1

**Published:** 2018-06-18

**Authors:** John J. Millichap

**Affiliations:** 1 Division of Neurology, Ann & Robert H. Lurie Children’s Hospital of Chicago, Chicago, IL; and Departments of Pediatrics and Neurology, Northwestern University Feinberg School of Medicine, Chicago, IL

**Keywords:** Neurology, Pediatrics, Child Development, Nervous System Diseases, Brain Diseases

## Abstract

In 2018, the mission of *Pediatric Neurology Briefs* (PNB) remains the same: “PNB is a continuing education service designed to expedite and facilitate the review of current scientific research and advances in child neurology and related subjects.”

In 2018, the mission of *Pediatric Neurology Briefs* (PNB) remains the same: “PNB is a continuing education service designed to expedite and facilitate the review of current scientific research and advances in child neurology and related subjects.” Recognizing that readers have accepted, and perhaps prefer, the online version of PNB the Editors have decided to take the transformation of the publishing model one step further. Beginning in 2018, PNB will publish the canonical version of the article online as soon as it has been accepted and typeset. The published articles will be collected into online volumes and an annual collection for print will be available at the end of every year.

PNB has been published since 1987 and consists of 31 volumes with greater than 3700 articles and includes over 10,000 citations referencing the works of approximately 28,000 scholarly authors. PNB was relaunched as an open access, peer-reviewed, journal with an expanded editorial board in January 2015. The Editors of PNB select source articles using criteria that include recent publication in a peer-reviewed journal and a topic of clinical value to practicing pediatric neurologists. Contributing Editors provide detailed summaries of published articles, followed by commentaries based on their experience and corroborated by appropriate supplementary citations. Also in 2015, PNB launched a new website and content management system capable of organizing peer-review and providing improved indexing, DOI assignment, and online full-text article view. Prior to the internet, the Editor periodically compiled and published the PNB articles in book form with index, according to subject heading and in chronological order [1-3].

In 2016, digitization of the 30 years of back issues was completed. There are now over 3700 full text open access articles available on the journal website. Also in 2016, PNB was selected for inclusion in PubMed Central^®^ (PMC). PMC is a free archive of biomedical and life sciences journal literature at the U.S. National Institutes of Health's National Library of Medicine (NIH/NLM). The two most highly accessed articles published in 2017 covered topics related to status epilepticus [4] and migraine [5].

The Editors would like to take this opportunity to sincerely thank the 2017 Contributing Editors for their effort and expertise (listed below in alphabetical order):

Nada Boutrid, MD

Melissa L. Cirillo, MD

Marytery Fajardo, MD

Tracy S. Gertler, MD, PhD

Kathleen M. Gorman, MD

Michael F. Hammer, PhD

Jonathan E. Kurz, MD

Divakar S. Mithal, MD, PhD

Juan A. Piantino, MD

Craig A. Press, MD, PhD

Hakim Rahmoune, MD

Garnett Smith, MD

Cynthia V. Stack, MD

Mark S. Wainwright, MD, PhD

Topic experts interested in contributing to PNB as authors or reviewers are invited to contact the Editor at j-millichap@northwestern.edu.

## Disclosures

The author has declared that no competing interests exist.
